# Trio sequencing in pediatric cancer and clinical implications

**DOI:** 10.15252/emmm.201708641

**Published:** 2018-03-05

**Authors:** Michaela Kuhlen, Arndt Borkhardt

**Affiliations:** ^1^ Department of Pediatric Oncology, Hematology and Clinical Immunology Medical Faculty Heinrich Heine University Düsseldorf Germany

**Keywords:** Cancer, Chromatin, Epigenetics, Genomics & Functional Genomics

## Abstract

In pediatric cancer, we advocate for trio sequencing of the child and its parents. This method can have substantial implications for cancer prevention in parents and siblings and even in more distant family members. It does not only help to identify a putative classical cancer predisposition syndrome in the index patient, but also detects the combinatorial effect of two independent risk variants in the same signaling pathway. This type of inheritance pattern could contribute to explaining the early occurrence of cancer in children and young adults and thereby inform early diagnosis, screening and preventive measures.

There are more than 100 known cancer predisposition syndromes (CPSs), including DNA damage repair defects, genetic instability syndromes, bone marrow failure syndromes, cell cycle and differentiation defects, transcription factors and pure familial leukemia syndromes, immunodeficiencies, and congenital/developmental syndromes (Table 1; Kuhlen & Borkhardt, 2015). Most of these CPSs are inherited in an autosomal dominant or compound heterozygous pattern; only a few are autosomal recessively or X‐linked transmitted. The most significant familial CPS is Li‐Fraumeni syndrome (LFS), which predisposes carriers to a 50% lifetime risk of developing cancer before the age of 30 and 90% risk before the age of 60. Affected patients are not only at high risk of developing secondary, treatment‐related cancers after irradiation or the use of alkylating agents, but also additional cancers unrelated to treatment. Early detection of these CPS—not just in patients but also in their close relatives—can therefore help to diagnose and treat tumors in the early stages. Villani *et al* (2016) demonstrated improved long‐term survival of carriers of a pathogenic *TP53* variant using a comprehensive surveillance protocol for early tumor detection. However, this assumes that every *TP53* carrier is identified early on, and not only after cancer diagnosis.

**Table 1:** List of cancer predisposition syndromes

Cancer predisposition syndrome (CPS)	Associated gene(s) (CPG)
**DNA repair disorders**	
Ataxia telangiectasia	*ATM*
Bloom syndrome	*BLM*
Fanconi anemia	*FANCA, FANCB, FANCC, FANCD1/BRCA2, FANCD2, FANCE, FANCF, FANCG, FANCI, FANCJ/BRIP1/BACH1, FANCL, FANCM, FANCN/PALB2, FANCO/RAD51C, FANCP/SLX4, FANCQ/XPF/ERCC4, FANCR/RAD51, FANCES/BRCA1, FANCT/UBE2T, FANCU/XRCC2, REV7/MAD2L2*
Nijmegen breakage syndrome	*NBN*
Rothmund–Thomson syndrome	*RECQL4*
Xeroderma pigmentosum	*DDB2, ERCC1, ERCC2, ERCC3, ERCC4, ERCC5, POLH, XPA*,* XPC*
Li‐Fraumeni syndrome	*TP53*
Constitutional mismatch repair deficiency	*MLH1, MSH2, MSH6, PMS2, EPCAM*
**Bone marrow failure/leukemia predisposing syndromes**	
Severe congenital neutropenia (Kostmann syndrome)	*ELANE, HAX1*
Constitutional thrombocytopenia	ANKRD26
MIRAGE syndrome	SAMD9
Ataxia‐pancytopenia syndrome	SAMD9L
Familial AML with mutated DDX41	*DDX41*
Congenital thrombocytopenia	*MECOM*
Bone marrow failure syndrome	*ERCC6L2*
Thrombocytopenia and absent radii syndrome	–
Congenital amegakaryocytic thrombocytopenia type I/II	*MPL*
Transcription factor	
Familial platelet disorder with propensity to myeloid malignancy	*RUNX1*
Familial AML	*CEBPA*
GATA2‐spectrum disorders	*GATA2*
Susceptibility to ALL	*PAX5*
Thrombocytopenia	*ETV6*
Ribosomal anomalies	
Diamond blackfan anemia	*RPS7, RPS10, RPS17, RPS19,RPS24, RPS26, RPL5, RPL11, RPL19, RPL35A*
Shwachman–Diamond syndrome	*SBDS*
Telomere maintenance	
Dyskeratosis congenita	*DKC1, TERC, TERT, TINF2, NHP2, NOP10, WRAP53*
**RASopathies**	
Neurofibromatosis type 1	*NF1*
Noonan syndrome	*PTPN11, SOS1, RAF1, RIT1, KRAS, NRAS, SHOC2*
Noonan syndrome with multiple lentigines	*PTN11, RAF1*
Capillary malformation–arteriovenous malformation syndrome	*RASA1*
Costello syndrome	*HRAS*
Cardio‐facio‐cutaneous syndrome	*BRAF, MAP2K1 (MEK1), MAP2K2 (MEK2)*
Legius syndrome	*SPRED1*
CBL syndrome	*CBL*
**Immunodeficiencies (by way of example)**	
Wiskott–Aldrich syndrome	*WAS*
PMS2 deficiency	*PMS2*
X‐linked lymphoproliferative syndrome	*SAP, XIAP*
IL2‐inducible T‐cell kinase deficiency	*ITK*
Ligase IV syndrome	*LIG IV*
DOCK8 deficiency	*DOCK8*
Cartilage hair hypoplasia	*RMRP*
**Familial cancer syndromes**	
Familial adenomatous polyposis syndrome	*APC, MUTYH*
Juvenile polyposis syndrome	*SMAD4, BMPR1A*
Peutz–Jeghers syndrome	*STK11*
MYTH‐associated polyposis	*MUTYH*
Lynch syndrome type	*MSH2, MSH6, MLH1, PMS2, EPCAM*
Multiple endocrine neoplasia type I	*MEN1*
Multiple endocrine neoplasia type IIA	*RET*
Multiple endocrine neoplasia type IIB	*RET*
Multiple endocrine neoplasia type IV	*CDKN1B*
Von Hippel–Lindau	*VHL*
Hereditary paraganglioma/pheochromocytoma syndrome	*SDHA, SDHB, SDHC, SDHD, SDHAF2, TMEM127, MAX*
Familial thyroid cancer	*RET, NTRK1*
Hyperparathyroidism‐jaw tumor syndrome	*CDC73*
PTEN hamartoma tumor syndrome	*PTEN*
Pleuropulmonary blastoma syndrome	*DICER1*
GLOW syndrome	*DICER1*
Nevoid basal cell carcinoma syndrome (NBCCS)/Gorlin syndrome	*PTCH1, SUFU*
Hereditary breast/ovarian cancer	*BRCA1, CHEK2, ATM, NBS1, RAD51, BRIP1, PALB2*
Rubinstein–Taybi syndrome	*CREBBP, EP300*
Schinzel–Giedion syndrome	*SETBP1*
NKX2‐1 syndrome	*NKX2‐1*
Hereditary leiomyomatosis and renal cancer syndrome	*FH*
Tuberous sclerosis complex (TSC)	*TSC1, TSC2*
Hereditary multiple exostoses	*EXT1, EXT2*
Kabuki syndrome	*KMT2D, KDM6A, MLL2*
Birt–Hogg–Dubé syndrome	*FLCN*
Neurofibromatosis type II	*NF2*
Schwannomatosis	*SMARCB1, LZTR1*
Meningeoma predisposition	*SMARCE1*
Non‐syndromic hereditary Wilms tumor	*WT1, CTR9*
Hereditary retinoblastoma	*RB1*
Hereditary neuroblastoma	*ALK, PHOX2B*
Malignant rhabdoid tumor syndrome	*SMARCB1, SMARCA4*
**Chromosomal abnormalities**	
Down syndrome/Trisomy 21	
Ullrich–Turner syndrome	
Trisomy 18	
rob(15;21)(q10;q10)c, ring chromosome 21	
Monosomy 7	
**Congenital/developmental disorders and overgrowth syndromes**	
Coffin–Siris syndrome	*SOX11, ARID1A, ARID1B, SMARCA4, SMARCB1, SMARCE1*
Nicolaides–Baraitser syndrome	*SMARCA2*
Bohring–Opitz syndrome	*ASXL1*
Mulibrey nanism	*TRIM37*
Beckwith–Wiedemann syndrome	–
Hemihypertrophy	–
Perlman syndrome	*DIS3L2*
Simpson–Golabi–Behmel syndrome	*GPC3, GPC4*
WAGR syndrome	–
Denys–Drash syndrome	*WT1*
Frasier syndrome	*WT1*
Weaver syndrome	*EZH2*
Sotos syndrome	*NSD1*
**Metabolic disorders**	
Citrullinemia	*SLC25A13*
Ornithine transcarbamylase deficiency	*OTC*
Argininosuccinate lyase deficiency	ASL
Arginase deficiency	*ARG1*
Familial pheochromocytoma and paraganglioma syndrome	*SDHA, SDHB, SDHC, SDHAF2*
Cowden syndrome 2	*SDHB*
Leigh syndrome	*SDHA, SDHB*
L‐2‐hydroxyglutaric aciduria	*L2HGDH*
Tyrosinemia	*FAH*

In the largest pediatric study to date, Zhang *et al* (2015) found an underlying CPS in 8.5% of childhood cancers, with *TP53* being the most commonly mutated gene. They used a tumor versus germline approach to analyze mutations in the affected children, which did not allow them to elucidate the ratio of CPSs caused by inherited versus *de novo* germline mutations in cancer predisposition genes (CPGs). Indeed, to determine the inheritance pattern and thus the risk of recurrence in other family members, a parent–child (trio) approach is needed (Fig 1A–E) as parents might be clinically unaffected owing to phenotypic variability, incomplete penetrance, gender‐specific cancer risk, and environmental exposure. The child's cancer diagnosis alone may already indicate a familial cancer predisposition and thus help to identify any cancer risk in siblings. In addition, identifying a familial predisposition offers the opportunity for early cancer surveillance in at‐risk family members. For instance, in children diagnosed with constitutional mismatch repair deficiency (CMMRD), an autosomal recessive CPS, transmitting parents are at risk of tumors on the Lynch syndrome spectrum including colorectal and endometrial cancer, which typically develop in the third decade of life (Taeubner *et al*, 2018b).

**Figure 1:**
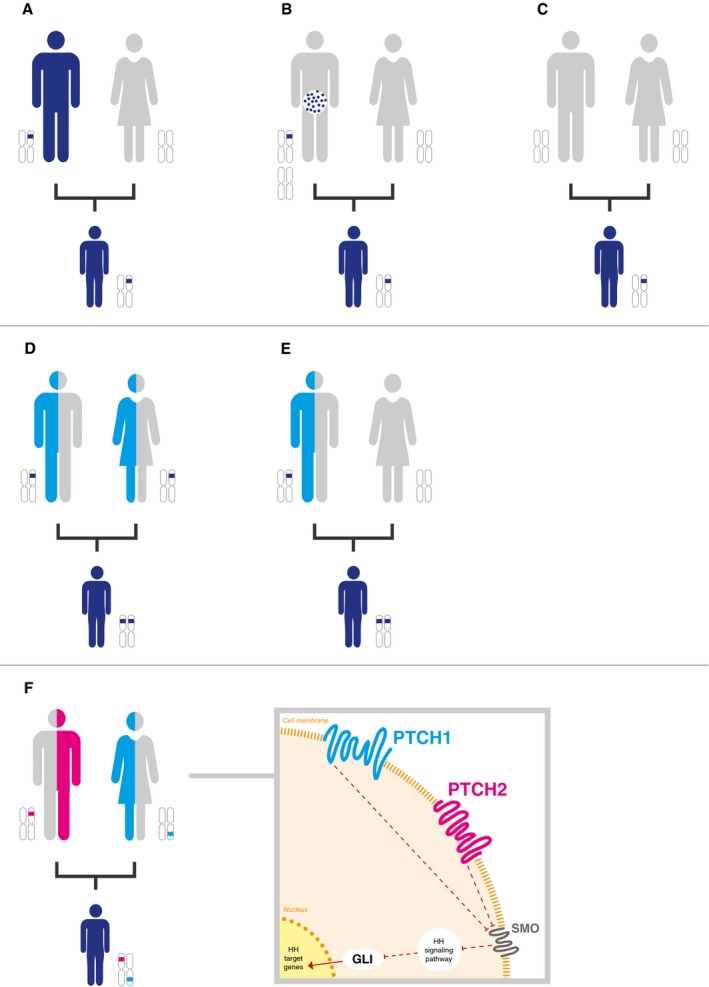
Inheritance patterns in children with cancer (A–C) Autosomal dominant inheritance—transmitted by the affected (or as yet clinically unaffected) father (A), transmitted by parental (in this case paternal) mosaicism (B), and originated *de novo* (C). (D, E) Autosomal recessive inheritance—transmitted by both unaffected parents (D) and one variant transmitted by an unaffected parent (in this case the father) and one originated *de novo* (E). (F) Concomitant digenic inheritance of two heterozygous variants exemplified by two germline variants in *PTCH1* and *PTCH2* in a newborn with congenital rhabdomyosarcoma, leading to activation of the sonic hedgehog signaling pathway. The *PTCH1* variant is inherited by the mother, while the *PTCH2* variant is inherited by the father. Both parents are clinically unaffected so far.

In our pediatric oncology department, we initiated a prospective study termed “Germline mutations in children with cancer”. Families whose child was newly diagnosed with cancer were offered a comprehensive whole‐exome sequencing (WES) of parent–child trios in combination with systematically collecting demographic, medical, and family history data. The study aimed to determine the interest in and acceptance of this approach in affected families, to analyze whether anamnestic data indicate a familial cancer predisposition, and to investigate an underlying CPS and its inheritance pattern. Notably, knowledge of a potentially underlying CPS, and particularly the risk of recurrence in other children, is of great interest to families who have a child diagnosed with cancer. Thus, the great majority of families (88.3%) opted for participation when we offered diagnostic trio WES sequencing (Brozou *et al*, 2018).

In addition to the most well‐known CPS such as LFS, neurofibromatosis, and Gorlin syndrome, we also identified a frequent genetic phenomenon characterized by the presence of at least two independent, monoallelic germline mutations in different genes involved in the same signaling pathway (Fig 1F). In these analyses, we set the threshold for single nucleotide variants (SNVs) to a minor allele frequency (MAF) of < 1% and a combined annotation‐dependent depletion (CADD) score of higher than 10. We only considered combined inherited SNVs to be potentially pathogenic if at least one *in silico* prediction tool classified the variant as likely to be damaging or deleterious. In addition, we defined that the mutations must either be inherited from the parents—one each from the mother and father—who were as yet clinically unaffected, or, alternatively, one SNV was transmitted from the mother or father, while the second SNV occurred *de novo* in the affected child. Such combined monoallelic double hits likely cause the clinical cancer phenotype by interrupting the affected signaling pathway. For example, one might speculate that combined germline mutations in *ATM* and *CHK1*, both playing a critical role in DNA damage repair, may alter *TP53* function and thus lead to a Li‐Fraumeni like‐phenotype that cannot be explained by *TP53* mutations alone. Significantly, such a phenomenon caused by inherited combined digenic low‐penetrance variants might present with clinically unaffected parents and an unremarkable family history.

Likewise, several observations in breast cancer patients led to the hypothesis that low‐penetrance cancer susceptibility polymorphisms act as modifier genes in *BRCA1*/*BRCA2* mutation carriers and non‐carriers to increase cancer risk. One could speculate that this involves genes that act as modifiers in the same CPG pathway, or low‐penetrance polymorphisms in *BRCA1*/*BRCA2* mutation non‐carriers (Smith *et al*, 2007; Polak *et al*, 2017). In fact, combined monoallelic mutations in Fanconi anemia/breast cancer (FA/BRCA) pathway genes have been identified in patients with a more severe disease phenotype. The FA/BRCA pathway plays an important role in the maintenance of genome integrity and is involved in the DNA damage response (DDR) and DNA repair pathways.

By trio WES, we identified two concomitant monoallelic germline mutations in *BRIP1* and *HIPK2* in an 11‐year‐old girl diagnosed with metastatic osteosarcoma (Fig 2). Mutations in *BRIP1*/*FANCJ* are associated with breast cancer, but, so far, not with osteosarcoma. Further *in silico* analysis predicted that the novel missense variant in *BRIP1*, which is located in the nuclear localization signal, is damaging and deleterious. Eventually, the mother, who transmitted the *BRIP1* variant, was diagnosed with breast cancer at the age of 46. *HIPK2*, which was transmitted by the father, is a crucial regulator of the DDR pathway and plays an important role in DNA double‐strand break repair.

**Figure 2:**
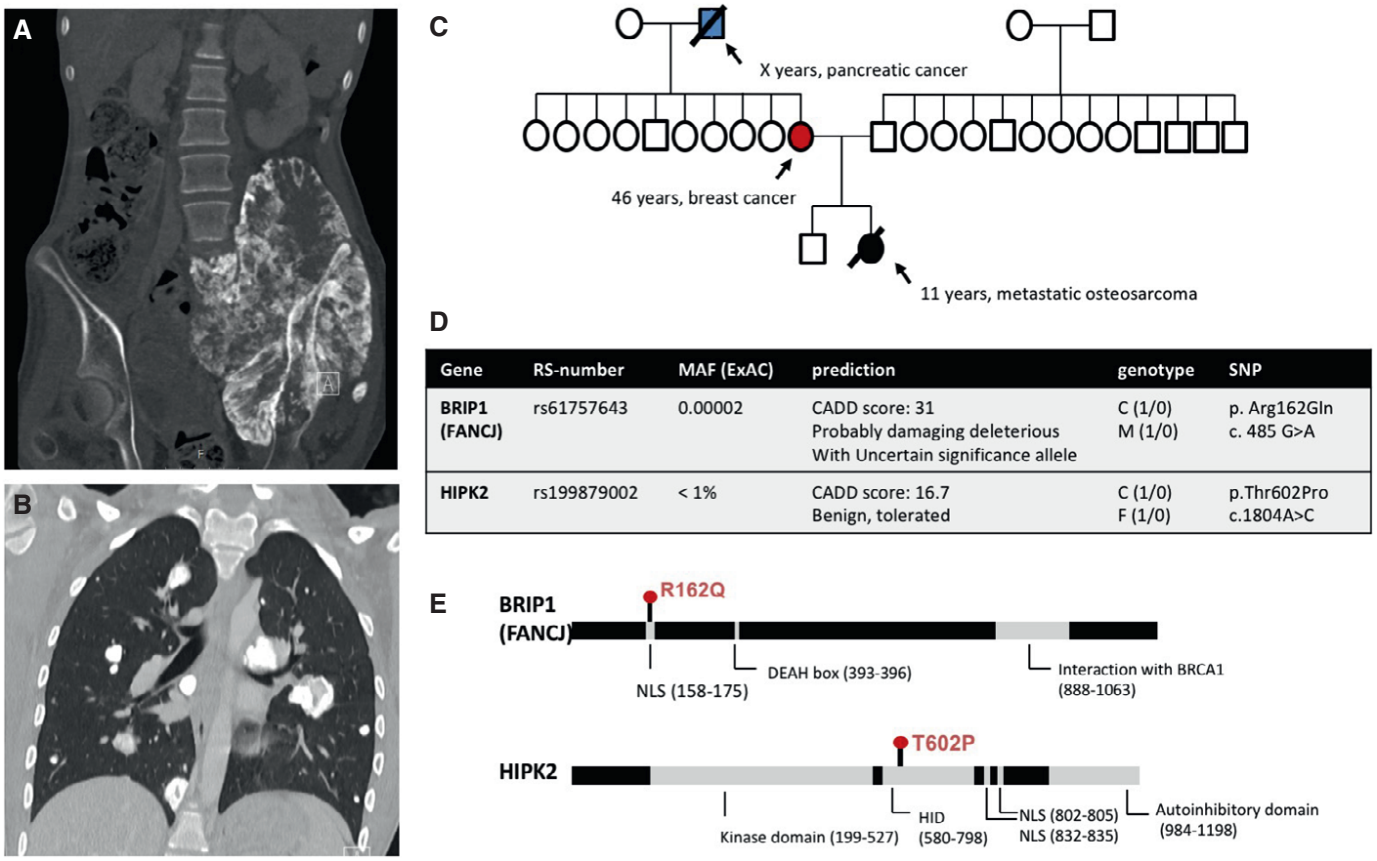
Patient with metastatic osteosarcoma and concomitant monogenic germline variants in *BRIP1* and *HIPK2* CT scans of the pelvic region (A) and lungs (B) which shows the large pelvic tumor growing into the spinal canal and multiple lung metastases; three‐generation pedigree of the family (C); WES of blood‐derived DNA from the patient and the parents revealed a novel missense variant (p.Arg162Gln) in the *BRIP1* gene located in the NLS (nuclear localization signal) inherited from the mother, and a missense variant (p.Thr602Pro) in the *HIPK2* gene inherited from the father (D); protein structure of BRIP and HIPK2 (E). The patient and parents were enrolled in our study termed “germline mutations in children with cancer”. This study was approved by the Ethics Committee of the Heinrich Heine University, Duesseldorf, Germany (Study Number 4886). Informed consent was obtained from the patient and both parents. Whole‐exome sequencing was performed on peripheral‐blood‐derived DNA in accordance with the WMA Declaration of Helsinki and the Department of Health and Human Services Belmont Report.

The data from our ongoing study suggest that such double hits are particularly frequent in the TP53 and FA/BRCA pathway. It is not clear yet to what extent such functional perturbations of key cancer pathways by at least two co‐inherited heterozygous digenic mutations from each parent appear in the germline of children with cancer. By way of example, we detected two concomitant heterozygous low‐penetrance germline variants in *PATCHED1* (*PTCH1*) and *PATCHED2* (*PTCH2*), two key sonic hedgehog (SHH) signaling pathway genes in a newborn with congenital embryonal rhabdomyosarcoma. Only the combination of these two mutations activated the SHH pathway, which may help to explain the very early onset of rhabdomyosarcoma in newborns. The parents, who transmitted one risk variant each, are clinically unaffected and did not show activation of the SHH pathway (Taeubner *et al*, 2018a).

We think that monoallelic, independent germline mutations in more than one CPG in the same cancer pathway should be considered pathogenic. Such double‐hit mutations, which are reminiscent of compound heterozygosity that causes many devastating Mendelian disorders—severe primary immunodeficiencies and metabolic disorders—are likely overlooked in the clinic if each is inherited by one clinically unaffected parent. Taking this inheritance pattern into account, we aimed to put it in a broader perspective, namely at the cancer pathway level (Fig 1F). However, it remains unclear to which extent this phenomenon may trigger or at least modify malignant transformation particularly in children, in whom, other than in adults, long‐term lifestyle factors are mostly negligible. Obviously, the likelihood of this phenomenon to occur purely by chance critically depends on the mutation load in the respective population and may vary across populations and genes.

In addition, whereas the pathogenicity of protein‐truncating mutations seems plausible, frequent missense variants may functionally be unimportant and found by chance if a given cancer pathway includes a sufficiently large number of genes. For instance, the Exome Aggregation Consortium (ExAC) database contains 567 missense variants (of any frequency) in *BRCA1*, 1186 in *BRCA2*, 46 in *RAD51*, and 385 in *PALB2*—genes involved in the FA/BRCA pathway—in 60,000 healthy individuals. On other hand, missense variants can even be more deleterious than truncations, if, for instance, the mutation exerts an additional dominant‐negative effect (di Masi, 2008). Hence, careful functional validation of identified variants is mandatory, but a tantalizing task in clinical practice. Contrary to what is commonly assumed, the functional alteration of a protein does not necessarily cause a complex clinical phenotype such as cancer in children. A complete understanding of this phenomenon will remain elusive until we can better characterize the role of protein‐altering genetic variation on cancer development. To this end, we need databases with functional analyses for different variants in each single gene.

For patients and their family members, the double hit‐one pathway phenomenon could have important clinical implications, including early diagnosis, assessment of cancer risk, and surveillance. In times of increasingly precise medicine, including early tumor detection and immunoprevention, this is of high relevance. The past 3 years have seen the development of early detection of premalignant lesions by analyzing circulating cell‐free DNA and molecular markers, clonal hematopoiesis by deep sequencing, combinatorial chemopreventive interventions, and new, FDA‐approved drugs and vaccines for cancer prevention (Kensler *et al*, 2016). Given these promising approaches, we expect novel options for cancer prevention and early detection to become available in the near future, along with surveillance programs such as in LFS. For instance, in families with CMMRD and Lynch syndrome, low‐dose aspirin is already recommended for preventing colorectal cancer (Burn *et al*, 2011). Trio sequencing therefore gives clinically important insights into inheritance patterns, the pathogenesis and mechanisms of cancer development in children, and provides a powerful tool to identify family members at risk. This method holds the promise of real precision cancer medicine including targeted prevention.

## Conflict of interest

The authors declare that they have no conflict of interest.
